# Fine-Scale Resolution of Runs of Homozygosity Reveal Patterns of Inbreeding and Substantial Overlap with Recessive Disease Genotypes in Domestic Dogs

**DOI:** 10.1534/g3.118.200836

**Published:** 2018-11-14

**Authors:** Aaron J. Sams, Adam R. Boyko

**Affiliations:** * Embark Veterinary, Inc., Boston, Massachusetts 02111; † Department of Biomedical Sciences, College of Veterinary Medicine, Cornell University, Ithaca, New York 14853

**Keywords:** inbreeding, inbreeding depression, identity-by-descent, canid

## Abstract

Inbreeding leaves distinct genomic traces, most notably long genomic tracts that are identical by descent and completely homozygous. These runs of homozygosity (ROH) can contribute to inbreeding depression if they contain deleterious variants that are fully or partially recessive. Several lines of evidence have been used to show that long (> 5 megabase) ROH are disproportionately likely to harbor deleterious variation, but the extent to which long *vs.* short tracts contribute to autozygosity at loci known to be deleterious and recessive has not been studied. In domestic dogs, nearly 200 mutations are known to cause recessive diseases, most of which can be efficiently assayed using SNP arrays. By examining genome-wide data from over 200,000 markers, including 150 recessive disease variants, we built high-resolution ROH density maps for nearly 2,500 dogs, recording ROH down to 500 kilobases. We observed over 678 homozygous deleterious recessive genotypes in the panel across 29 loci, 90% of which overlapped with ROH inferred by GERMLINE. Although most of these genotypes were contained in ROH over 5 Mb in length, 14% were contained in short (0.5 - 2.5 megabase) tracts, a significant enrichment compared to the genetic background, suggesting that even short tracts are useful for computing inbreeding metrics like the coefficient of inbreeding estimated from ROH (*F_ROH_*). In our dataset, *F_ROH_* differed significantly both within and among dog breeds. All breeds harbored some regions of reduced genetic diversity due to drift or selective sweeps, but the degree of inbreeding and the proportion of inbreeding caused by short *vs.* long tracts differed between breeds, reflecting their different population histories. Although only available for a few species, large genome-wide datasets including recessive disease variants hold particular promise not only for disentangling the genetic architecture of inbreeding depression, but also evaluating and improving upon current approaches for detecting ROH.

Chromosomal segments that are homozygous by descent (autozygous) are a hallmark of inbreeding. Close consanguineous matings typically result in offspring with several long runs of homozygosity (ROH), while matings between more distant shared relatives (as often occurs in small or bottlenecked populations) produce a distribution of ROH skewed toward shorter tract lengths (see, for example Figure 2 in (Howrigan *et al.* 2011)). Most organisms of interest contain a wide array of segregating, often rare, recessive or partially recessive variants that can produce a deleterious phenotype when exposed as homozygous on genomic segments of autozygosity. Therefore, the efficient and accurate identification of ROH is of immense interest in the field of genetics, particularly in conservation biology and plant/animal breeding where avoidance of inbreeding depression is of critical importance.

Because ROH are a direct consequence of inbreeding, the two concepts are closely related. Inbreeding is often estimated from pedigrees, where the coefficient of inbreeding (*F*) is calculated as half the coefficient of relatedness (*r*) between the parents of an individual (Wright 1922). However, a pedigree-based estimate of *F* merely measures the mean expected autozygosity of an individual and not the true inbreeding level for an individual—the actual proportion of the genome that is identical by descent—which depends on the actual segregation and transmission of chromosomal segments (Hill and Weir 2011; Keller *et al.* 2011). Furthermore, in many populations, pedigrees may be inaccurate, incomplete, or missing, leading to incorrect or biased estimates of inbreeding (Cassell *et al.* 2003).

Genetic marker-based *F* estimates can be more accurate than pedigree-based estimates, but estimates based on only a handful of markers are typically less precise than pedigree-based estimates. For example, early molecular approaches to indirectly estimate *F* from microsatellites involved calculations of multi-locus heterozygosity (MLH), *d^2^*, and internal relatedness (IR) (Coulson *et al.* 1998; Coltman *et al.* 1998; Amos *et al.* 2001; Slate and Pemberton 2002). However, several studies later demonstrated that small microsatellite panels are ineffective at accurately estimating *F* (Slate *et al.* 2004; Balloux *et al.* 2004; DeWoody and DeWoody 2005).

Dense genotyping from either whole-genome sequencing or array-based genotyping allows for the detection of ROH and the inference of autozygous segments of the genome. Long ROH are indicative of recent identity by descent (IBD) and the sum of these tracts is, in theory, the exact inbreeding level of an individual. However, what constitutes a “long” ROH is unclear (Peripolli *et al.* 2016) and establishing an optimum length threshold is a challenge (McQuillan *et al.* 2008).

The two parental chromosomes within a diploid individual can be considered IBD at any point, as IBD is ultimately determined by coancestry back to a coalescent event. Such a definition of IBD is unhelpful, of course, because every portion of the genome has a finite time to the most recent common ancestor and would be considered IBD under this definition. Defining a minimum ROH threshold length is thus necessary to create a meaningful IBD estimate and may be particularly important for studying genetic load since some evidence exists that longer tracts of homozygosity carry disproportionately more deleterious variation than shorter tracts (Szpiech *et al.* 2013). However, the threshold at which a ROH is considered as an IBD segment has generally been determined empirically (if not arbitrarily) as a tract length that is long enough to likely have been inherited from a recent common ancestry (Ku *et al.* 2010) and has not been determined by asking which length threshold is actually most useful for detecting ROH harboring deleterious recessive loci.

Estimates of inbreeding are correlated with negative fitness consequences and reduced breeding value of a wide variety of traits of interest in many populations (Lencz *et al.* 2007; Nalls *et al.* 2009; Szpiech *et al.* 2013; Zhang *et al.* 2015; Mészáros *et al.* 2015). Precise estimation of inbreeding is particularly important for understanding inbreeding genetic load (Charlesworth and Willis 2009). In particular, inbreeding genetic load must be traced to recessive deleterious variants harbored on actual ROHs, but the degree to which this load is a consequence of ROH of various sizes, and thus the thresholds by which biologically relevant IBD tracts should be inferred, is not well understood. Previous studies have used functional predictions rather than known deleterious mutations and have led to different conclusions as to whether short or long ROH harbor more deleterious genetic variation (Szpiech *et al.* 2013; Zhang *et al.* 2015).

Domestic dogs are an ideal organism in which to study the phenotypic effects of recent inbreeding. Hundreds of dog breeds, each with their own unique genetic history, form closed populations usually characterized by significant levels of autozygosity owing to founder effects, bottlenecks, popular sires, and artificial selection for conformation or performance. Approximately 200 known Mendelian recessive disease variants have been identified in dogs, the majority of which are potential models for human disease (Online Mendelian Inheritance in Animals) and can be tested efficiently with genotyping arrays. While a few of these variants, like the *SOD1* mutation, which predisposes dogs to degenerative myelopathy (a relatively late-onset disorder), are ancient mutations segregating in dozens of breeds, most of these disease variants are found in only one breed, or at most a few related breeds, suggesting they are relatively recent mutations (Awano *et al.* 2009; Boyko 2011).

If a method to detect ROH is highly accurate, the vast majority of homozygous recessive disease genotypes should occur in regions classified as ROH (and essentially all homozygous genotypes for diseases caused by recent mutations). This is because almost every disease variant is a consequence of a single mutation and thus occurs on a single genetic background. Therefore, all homozygous genotypes for that variant must occur in an ROH, at least until recombination breaks down the background haplotype so it is no longer detectable as ROH by genomic methods. Furthermore, the distribution of tract lengths for tracts overlapping these genotypes will enable a direct test of whether longer or shorter ROH tracts disproportionately harbor known recessive disease mutations. Even relatively long tract length thresholds (*e.g.*, 5 cM or 5 Mb) can capture shared ancestry ∼20 generations back which is substantially better than most pedigrees. However, even shorter thresholds capable of detecting coancestry even further back might be desirable. By incorporating knowledge of known recessive disease variants along with dense genome-wide data, we can better refine and evaluate methods of ROH detection, and more precisely investigate patterns of ROH across populations and across genomic regions.

Here, we estimate ROH using two popular methods, PLINK (Purcell *et al.* 2007; Chang *et al.* 2015) and GERMLINE (Gusev *et al.* 2009), to examine the association between ROH and known at-risk genotypes (observed cases of homozygous recessive deleterious genotypes) in domestic dogs. We hypothesize that at-risk genotypes will be highly enriched in ROH regions compared to the non-ROH genomic background. This enrichment can be used to evaluate the sensitivity and specificity of ROH-calling methods and can provide a direct test of whether longer ROH tracts are more or less enriched for these recessive disease variants. We additionally characterize the distribution of ROH in 11 common dog breeds as an example of how the distribution of ROH is influenced by the timing and extent of artificial selection in a breed.

## Materials and Methods

### At-Risk Dog Dataset

We queried Embark’s customer database on April 4^th^, 2018 for all dogs whose owners consented to participate in research that were homozygous (at-risk) for recessive deleterious conditions assayed by Embark’s platform. In total, we identified 678 at-risk cases in 670 dogs (some dogs were at-risk for more than one condition). We separated these at-risk cases into two categories: 1) at-risk for *SOD1*-based degenerative myelopathy (Awano *et al.* 2009), of which we observed 283 at-risk dogs, and 2) at-risk for all 28 other recessive deleterious conditions assayed by Embark, of which we observed 395 at-risk cases across 393 dogs (Tables S1, S2).

### Breed Dog Dataset

We queried Embark’s customer database on January 23^rd^, 2018 for all customer dogs identified as purebred by Embark from the most common 11 breeds and whose owners consented to participate in research. We then used the ‘–genome’ flag in PLINK v1.9 (Chang *et al.* 2015) to identify pairs of dogs for which the proportion of IBD (PI_HAT) was greater than 0.45 and used these pairs to remove dogs that were potentially related as parent-offspring or full siblings. In total, our final dataset included 1,792 dogs from 11 breeds (Table S3).

### Genotyping & Quality Control

Customer dogs were genotyped on Embark’s custom high-density genotyping platform containing approximately 220,000 markers including all 173,000 markers found on the Illumina CanineHD platform and probes to detect over 160 Mendelian disease variants. SNP filtering using PLINK 1.9 (Chang *et al.* 2015) was done to ensure genotype concordance rates above 99.99% and missingness rates below 0.1%. Genotype data were phased against a proprietary reference panel and missing data imputed using Eagle2 (Loh *et al.* 2016). SNP data were also pruned with PLINK to remove markers in close linkage disequilibrium using “–indep-pairwise 200 100 0.90”. After pruning, 170,728 autosomal and 4,395 chrX markers remained, for an average of one marker per 12.8 kb for autosomes (one marker per 28.2 kb on chromosome X).

### Defining Runs of Homozygosity with PLINK

We generated ROH for at-risk dogs in PLINK using software version 1.9 (Chang *et al.* 2015) (which uses the algorithm from software version 1.07 (Purcell *et al.* 2007)).

 –homozyg-window-het 0
 –homozyg-snp 41
 –homozyg-window-snp 41
 –homozyg-window-missing 0
 –homozyg-window-threshold 0.05
 –homozyg-kb 500
 –homozyg-density 5000 (set high to ignore)
 –homozyg-gap 1000 (set high to ignore)

### Defining Runs of Homozygosity with GERMLINE

Initially, we attempted to use GERMLINE’s internal filtering to identify ROH >= 0.5 Mb and consisting of at least 41 markers using the following parameters:

germline -homoz-only -min_m  0.5 -err_hom 0-err_het 0 -bits 41 -w_extend

where -min_m = 0.5 is in units of Megabase-pairs (all measurement in this study was computed in physical distance).

However, we noticed an issue with the germline software in which using the *-w_extend* flag in conjunction with the *-homoz-only* whereby all tracts are extended beyond the first mismatching marker to the end of the next slice (or beginning of the previous slice).

As an alternative, we used the following command in germline to generate preliminary homozygosity tracts for all dogs in this study:

germline -homoz-only -min_m 0.5 -err_hom 0 -err_het 0 -bits 1 -w_extend

This identified all segments of the genome >500 kb with no heterozygous markers. We then merged all such segments separated by <50 kb from a neighboring autozygous segment and subsequently removed all merged segments containing fewer than 41 markers (to avoid spurious inference of ROH in regions with few markers). We found this approach superior to allowing a certain set number of heterozygous markers within an ROH for two reasons: (1) requiring no heterozygous variants for at least 500kb vastly improves the specificity for detecting short ROH (500kb - 4000kb), and (2) allowing one or a number of tightly clustered (<50kb) heterozygous variants between two ROHs improves the sensitivity for detecting long ROHs that would otherwise be broken up by genotyping error or copy-number variation (deletions or duplications, most of which are <50kb, can lead to clustered heterozygous at the markers within the structural variant).

### Defining *F_ROH_*

*F_ROH_* was computed as in previous studies (*e.g.*, McQuillan *et al.* 2008) as:

$$F_{ROH_{j}}\;=\frac{\sum_{k}^{}\text{length}(ROH_{k})}{L};$$

where *ROH_k_* is the *k*th ROH in individual *j*’s genome and *L* is the total length of the genome (or X-chromosome).

### Data Availability

File S1 contains a list of all supplemental files. File S2 contains supplemental figures, tables, and references. File S3 contains phenotype information for at-risk dogs in this study. File S4 contains breed and sex information for breed dogs in this study. Files S5 and S6 contain genotype and marker location information in *PLINK* .ped/.map format for at-risk dogs in this study. Files S7 and S8 contain genotype and marker location information in *PLINK* .ped/.map format for breed dogs in this study. File S9 contains a python script for postprocessing germline homozygosity tracts generated using the flags presented in Materials & Methods. Supplemental material available at Figshare: https://doi.org/10.25387/g3.7330151.

## Results

### Distribution of overlaps of ROH with known homozygous at-risk alleles

In total, 670 dogs whose owners consented to participate in research were homozygous for at least one of 29 Mendelian disease alleles assayed by Embark and considered in our analysis. Of those, we measured the genome-wide distribution of ROH in order to compare it to the set of ROH overlapping a homozygous at-risk allele in our sample. We identified the set of ROH overlapping each occurrence of an at-risk genotype and summarized these tracts in four length categories (< 0.5 Mb–below ROH detection threshold; 0.5 Mb - 2.5 Mb–short; 2.5 Mb - 5.0 Mb–medium; > 5Mb–long).

We analyzed ROH generated from PLINK and GERMLINE using similar parameters for identifying ROH. In short, we considered ROH >= 0.5 Mb as long as they consisted of at least 41 markers. The results are broadly consistent between these two analyses (Table 1), although GERMLINE was modestly more sensitive, in terms of at-risk genotypes overlapping GERMLINE ROH tracts slightly more often than PLINK-generated ROH tracts, so we focus on results from the former here.

**Table 1:** Analysis of runs of homozygosity (ROH) in dogs carrying homozygous recessive deleterious mutations. ROH > 0.5 Mb (detected by our ROH analysis) harbor recessive deleterious alleles at minimum 29.8X more than ROH < 0.5 Mb

	All ROH Tracts**		ROH Harboring *SOD1*		ROH Harboring other recessive deleterious mutations		Relative risk compared to ROH < 0.5 Mb	
ROH Length	PLINK	GERMLINE	PLINK	GERMLINE	PLINK	GERMLINE	PLINK	GERMLINE
< 0.5 Mb*	75.9	75.1	36.4	34.3	9.9	7.8	1.0	1.0
0.5 - 2.5 Mb	4.5	4.7	19.4	18.7	14.9	14.7	25.7	29.8
2.5 - 5.0 Mb	3.3	3.1	15.2	14.5	11.6	11.4	27.4	35.7
> 5.0 Mb	16.4	17.1	29.0	32.5	63.5	66.1	29.8	37.0

* ROH below our detection threshold.

** Average fraction of genome composed of each tract length category across all dogs.

**Figure 1:**
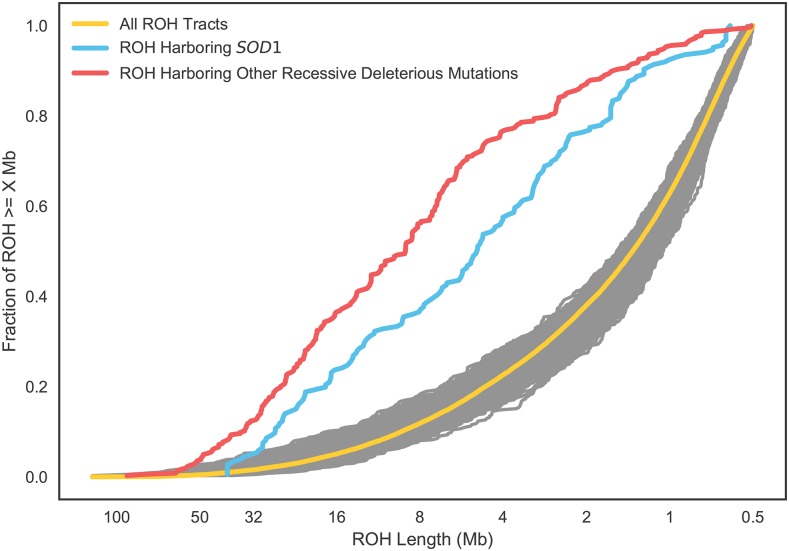
Cumulative density plot of runs of homozygosity (ROH) by length. Tracts are ordered from longest to shortest. Yellow line is all ROH in all dogs at-risk for a deleterious recessive disease, excluding the *SOD1* Degenerative Myelopathy (DM) allele. Red line is the distribution of ROH harboring homozygous recessive deleterious genotypes (excluding *SOD1*) in the same set of dogs. Blue is the set of ROH harboring homozygous genotypes of the *SOD1* DM allele only. Gray lines are 1000 sets of ROH, with the same sample size as the red line, sampled randomly from the set of all ROH and illustrate that the blue and red sets of ROH are highly non-random samples from the full set of ROH.

For all at-risk cases excluding *SOD1*, 92% of at-risk genotypes had an ROH overlapping the at-risk allele. While the longest ROH in our analysis harbor the majority (66%) of deleterious recessive alleles in this panel of at-risk dogs, short ROH nonetheless harbor known recessive disease homozygous genotypes at a rate nearly 30x higher than stretches of DNA that are not considered ROH. Across all tract lengths that we considered, the relative risk of a ROH carrying a deleterious mutation was similar across classes, suggesting that ROH of all lengths may contribute to inbreeding depression in dogs (see Figure 1, Table 1). To ensure that the deviation of the tracts overlapping at-risk genotypes is not a product of random sampling, we resampled the full distribution of homozygosity tracts from at-risk dogs to mimic the sampling of tracts from at-risk dogs (see Figure 1).

Although all the recessive diseases we studied had at-risk genotypes that were highly enriched in ROH tracts, the enrichment was not uniform across these disease variants. Notably for the *SOD1* mutation leading to canine degenerative myelopathy (DM) (Awano *et al.* 2009), an ancient mutation found in dozens of breeds, we observe a weaker enrichment of at-risk genotypes in long ROH (Figure 1, Table 1).

### Genome-wide distribution of ROH in 11 common dog breeds

**Figure 2:**
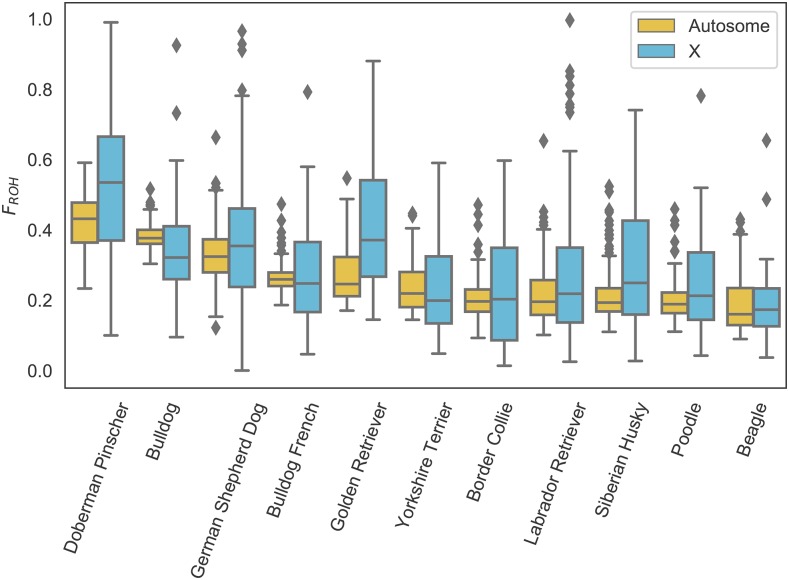
Distribution of *F_ROH_* in 11 common dog breeds. Breeds ordered from highest mean autosomal (yellow) inbreeding to lowest. Inbreeding in females only for chromosome X plotted in blue.

We estimated and analyzed the distribution of ROH in 1,792 dogs from 11 common dog breeds. First, we calculated *F_ROH_* for all breed dogs and assessed the distribution within breeds for both autosomes and chromosome X. Of the breeds we analyzed Doberman Pinscher had the highest overall levels of *F_ROH_* and Beagle the lowest. In general, *F_ROH_* of chromosome X varied in concert with the autosomes, although some breed (*e.g.*, Doberman Pinscher and Golden Retriever) had somewhat elevated *F_ROH_* on X compare to autosomes while others (*e.g.*, Bulldog) had lower *F_ROH_* (Figure 2).

**Figure 3:**
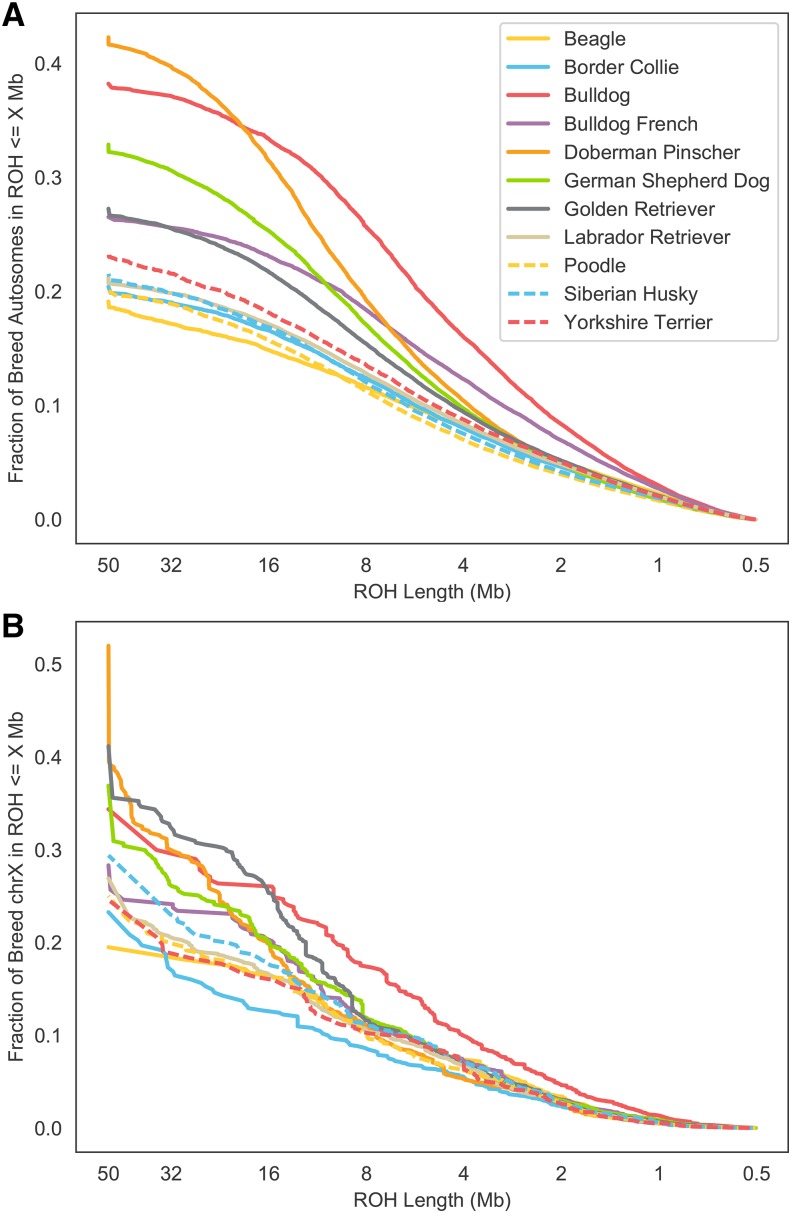
Cumulative density of runs of homozygosity (ROH) in 11 common breeds. A) Distribution of autosomal ROH across breeds. B) Distribution of chromosome X ROH in females only across breeds.

For each breed, we also assessed the distribution of ROH by length across all dogs in the breed (Figure 3). These distributions illustrate variation in the timing of diversity loss via inbreeding across breeds. For example, while today Doberman Pinschers have the highest average *F_ROH_* of all breeds in our analysis, the relatively higher fraction of inbreeding in short tracts in Bulldogs reflects the tremendous bottleneck that occurred in that breed after bull-baiting was banned in the 1830s and the breed was driven to the brink of extinction (Pedersen *et al.* 2016). Finally, for each breed we calculated a map of the local density of ROH, in other words the fraction of dogs in the breed sample carrying a ROH at each position (Figures S1-11). These maps highlight the deterministic loss of diversity (ROH islands) within breeds, and variation in ROH islands across breeds. Regions of homozygosity associated with fixation of certain variants (*e.g.*, the chr13 *RSPO2* locus in poodles) are clearly evident and are concordant with previously identified homozygosity regions in these breeds (c.f. (Vaysse *et al.* 2011). However there is also marked variation in rates of homozygosity outside of these fixed haplotype windows, demonstrating that drift and selection have led to non-uniform diversity loss across the genome in these breeds.

## Discussion

Our study is the first to examine patterns of ROH in dogs to a resolution of 500kb. While undoubtedly the sensitivity and specificity of detecting ROH is lower in short (0.5 - 2.5 Mb) tracts compared to long ones, particularly in regions of the genome with low marker density and/or high recombination rates, we observe a clear signal of enrichment of known recessive deleterious homozygous genotypes in these regions (Figure 1, Table 1). Homozygous recessive deleterious genotypes are consistently enriched 30-37-fold in ROH compared to the non-ROH genetic background regardless of ROH tract length. Thus, short tracts on aggregate represent a real signal of autozygosity, and furthermore these short tracts, often overlooked in studies of ROH, may be important and measurable contributors to inbreeding depression in dogs and other species. Indeed, 4.8–10.7% of the genomes of these 11 common breeds were covered by ROH tracts between 0.5 to 2.5 Mb (Figure 3A) and these tracts contained 18.7% of the *SOD1* and 14.7% of the non-*SOD1* known deleterious recessive homozygous genotype calls. Only 34.3% and 7.8% of the *SOD1* and non-*SOD1* homozygous genotype calls were not detected in ROHs, respectively (Table 1). To determine whether gaps in ROH introduced by genotyping error may explain some cases of these false negative cases, we repeated our analysis, doubling the gap filling parameter from 50 to 100kb, and found that no additional homozygous recessive deleterious genotypes were found in ROH. Because these false negative calls were usually flanked by many homozygous markers (but for less than 500kb) rather than heterozygous markers, we believe they are due to ROH below our detection threshold and not due to point mutations arising on different haplotype backgrounds.

Knowing the extent to which long *vs.* short ROH contribute to inbreeding depression would enable an assessment of the risk posed by recent consanguineous matings, which are often avoidable in dog breeding, and the risk from matings between more distant relatives which is generally unavoidable for purebred dogs. Because large-effect deleterious loci are purged by selection over time, segregating large-effect variants tend to be younger than neutral or weakly deleterious variants, and thus are likely to be breed-specific like most of the Mendelian disorders in this study). Therefore, if inbreeding depression in dogs is mainly caused by rare, large-effect recessive (or partially recessive) variants, the contribution of long *vs.* short tracts to inbreeding depression is likely well approximated by the contribution of long *vs.* short tracts to recessive homozygotes at known Mendelian disease alleles, most of which are rare on aggregate, although possibly common in the breed(s) they affect. In contrast, if inbreeding depression is mainly caused by common, small-effect recessive or partially recessive variants, the contribution of short tracts to inbreeding depression will be much greater, possibly even greater than the contribution of short tracts to DM risk at the *SOD1* locus. Further studies examining the distribution of ROH for different types of deleterious recessive loci (*e.g.*, Mendelian *vs.* complex, or strong *vs.* weak) in actual or in silico populations are needed to address this, and the results may depend strongly on the demographic history of the population and the specific phenotype(s) being used to measure inbreeding depression.

Within dogs, we see substantial variability in levels of autozygosity between breeds as well as across the genome within a breed. These differences represent the unique history of each breed, and the effect of drift and selection for particular traits over time. At one extreme, many breeds have complete autozygosity in certain windows of the genome. However, genomic regions of high autozygosity that are not completely fixed are also evident in every breed, and efforts to preserve breed diversity should focus on preserving rare haplotypes in these regions rather than rare markers in randomly selected genomic regions. Where fixed haplotypes harbor deleterious variation, marker-assisted crossings and backcrossings to introduce new diversity at the locus or gene-editing techniques like CRISPR/Cas9 to remove the deleterious variant(s) are required to rid a breed population of harmful variants.

Given the intense interest in developing and comparing methods to detect ROH, it is somewhat surprising that these methods are not typically evaluated for their sensitivity and specificity to detect known deleterious recessive mutations (indeed we are aware of no other genomic studies that have done so). While several studies have examined predicted deleteriousness of variants (Szpiech *et al.* 2013; Zhang *et al.* 2015), current methods for predicting deleteriousness are almost certainly less accurate than current methods to detect ROH (and predicting recessiveness is even more fraught), making them a poor way to evaluate the sensitivity and specificity of ROH detection methods. Being able to evaluate ROH methods in this way, however, is extremely valuable for comparing ROH detection methods and fine-tuning parameters to optimize accurate ROH inference for a population and genomic dataset of interest. Accurate ROH tract detection is invaluable not only for inferring the coefficient of inbreeding and other genetic parameters of interest, but also for accurate reconstruction of population history and identification of relatives.

The dog is an excellent genomic model for evaluating ROH methods as over 200 Mendelian, mostly recessive, variants are known and the requisite genomic resources (*e.g.*, high-quality reference genome and high-density SNP arrays) are available, although at present only the canine array platform used in this study includes both dense genome-wide coverage and probes to assay most of the Mendelian variants known in dogs. Although humans generally have much lower levels of inbreeding than purebred dogs, many more recessive disease variants are known in humans (Online Mendelian Inheritance in Man), and even denser arrays (including probes for many Mendelian variants) have been used to investigate over 10 million humans to date, largely on commercial DNA testing platforms (https://isogg.org/wiki/Autosomal_DNA_testing_comparison_chart). Thus, high-powered studies looking at many more at-risk loci and many more at-risk individuals with even denser marker panels could potentially be done to further investigate the sensitivity and specificity of various ROH methods and the contribution of different size ROH tracts to inbreeding risk if privacy concerns could be managed and data access granted to researchers in the field. Until then, researchers are encouraged to use this publicly available canine genetic database and to develop similar databases in other model genetic species to improve both the methodology by which ROH is computed and our insights into the genetic architecture of inbreeding depression in these species.
